# Protocol for a Trial to Assess the Efficacy and Applicability of Isometric Strength Training in Older Adults with Sarcopenia and Dynapenia

**DOI:** 10.3390/healthcare13131573

**Published:** 2025-07-01

**Authors:** Iker López, Juan Mielgo-Ayuso, Juan Ramón Fernández-López, Jose M. Aznar, Arkaitz Castañeda-Babarro

**Affiliations:** 1 Department of Health Sciences, Faculty of Health Sciences, University of Burgos, 09001 Burgos, Spain; coordinadorfc@kirolene.net (I.L.); jfmielgo@ubu.es (J.M.-A.); 2 Kirolene, San Ignacio Auzunea 5, 48200 Durango, Spain; r.fernandez@kirolene.net; 3 Baigene S.L., 01510 Alava, Spain; josem@baigene.com; 4 Health, Physical Activity, and Sports Science Laboratory, Department of Physical Activity and Sports, Faculty of Education and Sport, University of Deusto, 48007 Bizkaia, Spain

**Keywords:** sarcopenia, dynapenia, isometric resistance training, traditional resistance training, type II fibres

## Abstract

**Background:** Sarcopenia (loss of muscle mass) and dynapenia (loss of strength) are prevalent in older adults aged 70 years and over. Both have an impact on their functional ability and quality of life, with type II muscle fibres being particularly affected. Although traditional resistance training (TRT) is effective, it presents technical difficulties and an increased risk of injury among this vulnerable population. Isometric strength training (IST) is a potentially safer, more accessible and more effective alternative. **Objective:** To describe the protocol of a single-arm, pre-post intervention trial designed to evaluate the efficacy and applicability of a 16-week IST programme on muscle strength, skeletal muscle mass, quality of life and applicability (safety, acceptability, perceived difficulty) in 18 older adults aged 70 years and above with a diagnosis of sarcopenia and dynapenia. The influence of genetic and environmental factors on the variability of response to IST will also be explored. **Methodology:** The participants, who have all been diagnosed with sarcopenia according to EWGSOP2 (European Working Group on Sarcopenia in Older People 2) criteria, will perform two IST sessions per week for 16 weeks. Each 30-min session will consist of one progressive set (total duration 45 s to 90 s) for each of the eight major muscle groups. This series will include phases at 20% and 40% of individual Maximal Voluntary Isometric Contraction (MVIC), culminating in 100% Maximal Effort (ME), using the CIEX SYSTEM machine with visual feedback. The primary outcome variables will be: change in knee extensor MVIC and change in Appendicular Skeletal Muscle Mass Index (ASMMI). Secondary variables will be measured (other components of sarcopenia, quality of life by EQ-5D-5L, use of Likert scales, posture and physiological variables), and saliva samples will be collected for exploratory genetic analyses. The main statistical analyses will be performed with *t*-tests for related samples or their non-parametric analogues. **Discussion:** This protocol details a specific IST intervention and a comprehensive evaluation plan. The results are expected to provide evidence on the feasibility and effects of IST among older adults with sarcopenia and dynapenia. Understanding individual variability in response, including genetic influence, could inform the design of more personalised and effective exercise strategies for this population in the future.

## 1. Introduction

Ageing brings with it biological changes which, even in the absence of disease, lead to a progressive loss of muscle mass (sarcopenia), strength (dynapenia) and musculoskeletal function [1]. Sarcopenia has been defined by the European Working Group on Sarcopenia in Older People (EWGSOP) [2,3] as low muscle mass and strength and/or poor physical performance. It has a prevalence of 1–29% in older adults and may exceed 50% in institutionalised populations or those with comorbidities [1,2,4]. Dynapenia (loss of strength) often precedes sarcopenia, has a greater impact on functional ability, and is a robust predictor of falls, disability and mortality [1,2,4]. This muscle loss, which begins as early as age 30, accelerates after age 60 [5], with muscle tissue decreasing by 2.1% per year from age 50, while strength decreases by 1.5% (50–60 years) and up to 3% thereafter [6]. These processes are linked to impaired protein synthesis [7,8,9], chronic inflammation [10] and muscle disuse [1]. As a consequence, there is a reduction in physiological resilience, quality of life and mobility. This is coupled with an increased risk of falls [1], diabetes [11], osteoporosis [12] and cognitive impairment [13,14,15], which can lead to growing levels of morbidity and mortality.

Physiologically, sarcopenia and dynapenia mainly affect type II (fast) muscle fibres [16,17,18]. Their loss is associated with hormonal declines (testosterone, GH, IGF-1) [19,20,21] and increased inflammatory cytokines (TNF-α, IL-6), which contribute to systemic inflammation and muscle degeneration [18,20,22]. Strength training (ST) is a key tool to prevent muscle inactivity, sarcopenia and dynapenia [1,23,24], improving not only physiological frailty but also physical function, mobility, independence, chronic disease management, psychological well-being and quality of life [23,25,26].

Stimulation of type II fibres is crucial, but requires high intensities of effort [27,28,29,30]. Traditional resistance training (TRT), while effective when following recommended guidelines (2–3 sets of 6–12 repetitions at 70–85% of 1 RM, performed 2–3 times per week) [1] and incorporating proposed power exercises (using moderate loads at high speeds, typically 40–60% of 1 RM), presents certain challenges: high-threshold motor units are only activated near muscular failure [31,32], which increases the risk of compensatory movements and potential injuries [1]. In addition, high-speed, moderate-load exercises require high technical control and may injure various joints [33,34,35].

In this context, isometric strength training (IST) is a valuable alternative, used in rehabilitation, physical training and special populations [36,37,38,39]. Maximal voluntary isometric contraction (MVIC) is a reliable measure of health and sports performance [39,40,41]. IST has been shown to be effective against sarcopenia and dynapenia, inducing improvements in strength and hypertrophy [42,43,44,45]. Movement intention can be as effective as contraction velocity in improving Rate of Force Development (RFD) [39,46], and mechanical strain at maximal IST can exceed that of maximal concentric actions [36,47], optimising stimulation of high-threshold fibres.

There are mainly two types of isometric action: Pushing Isometric Muscle Action (PIMA) (push/pull against immovable resistance) and Holding Isometric Muscle Action (HIMA) (static load maintenance) [48]. Variations of IST include modification of joint angles [39,49,50], intensity or duration of contraction [39,51,52]. PIMA at Long Muscle Length (LML) appears to maximise mechanical tension [37,53] and promote early activation of high-threshold motor units [46,54]. Training at LML has shown superiority for hypertrophy and changes in muscle architecture (increased fascicle length) [44,50,55]; in fact, training at LML and Middle Muscle Length (MML) generates greater improvements than at Short Muscle Length (LMS) [44,45,50]. IST protocols of longer duration (e.g., 4 × 30 s) also appear to induce greater hypertrophy than those of shorter duration (e.g., 4 × 10 × 3 s) with equal time under tension [51]. In addition, IST allows controlled, pain-free work, preventing injury [36,37,39,56] and having acute analgesic effects [57,58] mediated by corticomotor pathways [38]. Thus, IST is a viable and safe alternative to TRT in older adults, although its efficacy may vary on an individual basis.

The variability in response to training and progression of sarcopenia may be partially explained by genetic factors. Previous studies have associated polymorphisms with sarcopenia (ACTN3; MTHFR; NRF2) [59,60], muscle hypertrophy (TGFβ; Myostatin, GDF-8) [61,62] and response to ST (ACTN3, ACE, NOS3, PPARGC1A/B, VEGFA) [63,64,65,66,67,68,69]. Analysing these factors together with others related to inflammation (CRP, IL6) [70,71], oxidative damage (SOD2, CAT, GPX), cell damage/regeneration (CASP, MMP3, GDF5, TNC) [71,72,73] and nutrigenomic factors (FAP2, ADBR, UCP2, GCKR, ADCY5, GIPR, HFE, TF) [74,75,76] could help to understand variability following the proposed intervention.

Considering the impact of sarcopenia/dynapenia, the difficulties of TRT and the potential of IST, there is a clear need to design and rigorously evaluate specific and optimised IST protocols for older adults, investigating their efficacy and applicability, as well as the influence of individual factors.

Therefore, the main research question that this protocol seeks to address is whether a 16-week IST programme can be an effective, safe and applicable intervention to counteract sarcopenia and dynapenia and improve quality of life in older adults, and to explore the individual factors that modulate response. Although IST is generally considered safe, potential discomfort or adverse events will be carefully monitored, as detailed in the methodology section (see Section 2.2.2 on safety assessment).

Consequently, the main objective of this study is to design an intervention protocol to evaluate the effect of 16 weeks of IST on sarcopenia, dynapenia and quality of life among adults aged 70 years and above. In addition, the influence of genetic and environmental variables on the variability of response to IST will be analysed in order to optimise its applicability and efficacy in this population group.

With a view to meeting the main objective of the study, a protocol is proposed to achieve the following strategic objectives:

1. To assess the reduction of sarcopenia and dynapenia by increasing muscle mass and strength following the application of an IST protocol in older adults.
2. To analyse the improvement in quality of life following the application of the IST protocol.
3. To assess the applicability of the IST in terms of safety for, and acceptability and perceived difficulty by older adults.
4. To determine the influence of genetic factors on the response to IST, considering variables related to muscle type, fibre damage and regeneration, vascularisation, inflammation, resistance to oxidative damage, and fat and carbohydrate metabolism.

The main hypothesis of the study is that the IST represents a highly effective choice for the reduction of sarcopenia and dynapenia, contributing to the improvement of health and quality of life among older adults. In addition, the following secondary hypotheses are proposed:

 **H1.** *IST has a low risk of injury in older adults.*

 **H2.** *IST increases the acceptance of strength training by older adults.*

 **H3.** *IST is perceived as technically simple by older adults.*

 **H4.** *The response to IST is modulated by genetic factors associated with muscle type, inflammation, tissue regeneration and energy metabolism.*

## 2. Methodology

### 2.1. Design of the Intervention Study

This non-randomised, single-group, pre-post-intervention, exploratory trial will evaluate the impact of IST on sarcopenia and dynapenia in adults over 70 years of age across a 16-week period. A detailed timeline of enrolment, interventions, and assessments is presented in Table 1. Each participant will act as their own control, comparing their baseline and post-intervention status. A concurrent comparison group will not be included in this phase, which will focus on feasibility and preliminary effects (see Section 4 for a discussion of limitations). Allocation to different intervention groups is not applicable. The study aims to measure the effectiveness of IST in improving muscle mass, strength and quality of life, as well as its practical applicability. Participants will be recruited in Vitoria (Alava) and Zamudio (Bizkaia, Spain) through Baigene S.L., the Judizmendi Civic Centre (Vitoria-Gasteiz City Council) and the Estadio Vital Fundazioa Foundation.

**Table 1:** Timeline for Study Participation.

Study Phase	Main Activities	Approximate Timing
Recruitment	Identification of potential participants, initial contact through the collaborating organisations (BAIGENE S.L., Judizmendi Civic Centre, Fundación Estadio Vital Fundazioa).	Prior to the start of the study
	1st Meeting (Information and Consent): Detailed explanation of the study, objectives, exercises; signing of the informed consent form.	Approx. 2–3 weeks before baseline assessment
	2nd Meeting (Criteria Evaluation): Questionnaire (sociodemographic, habits, pathologies, medication); Comprehensive Geriatric Assessment (CGA); Review of analytical data; Anthropometric measurements and Body Composition (height, weight, BMI, InBody for SMM/ASMM); Diagnostic tests for sarcopenia (gait speed, grip strength, and confirmation of low muscle mass with ASMM/ASMI) according to EWGSOP criteria.	Approx. 1–2 weeks before baseline assessment
Baseline Assessment	(Pre-Intervention Assessment): Measurement of physiological variables (BP, HR, SpO2), functional capacity (MVIC of 8 muscle groups, static posture), quality of life (EQ-5D-5L), saliva sampling for genetic analysis.	Week 0 (one week before the start of the IST)
Intervention	IST Programme: 2 sessions/week, 30 min/session, supervised. Progression according to Table 2	Weeks 1 to 16
	Continuous monitoring: Record of attendance, protocol compliance, adverse events (see Section 2.4.3), medication and lifestyle changes (see Section 2.1.3).	Weeks 1 to 16
Final Evaluation	(Post-Intervention Evaluation): Repetition of all baseline measurements (except genetic analysis), repetition of sarcopenia diagnostic tests to assess changes; applicability questionnaires (safety, acceptability, difficulty).	Week 17 (one week after the end of the 16-week IST)
Closing of the Study	Final data analysis, preparation of reports and scientific publications.	After Week 17

**Table 2:** The 16-week IST protocol.

Weeks	Total Working Time	Part 1		Part 2		Part 3	
		Time	%MVIC	Time	%MVIC	Time	%ME
1–4	45″	15″	20	15″	40	15″	100
5–8	60″	20″	22	20″	42	20″	100
9–12	75″	25″	24	25″	44	25″	100
13–16	90″	30″	26	30″	46	30″	100

#### 2.1.1. Criteria for Inclusion/Exclusion in Research

##### Sampling Method

Consecutive non-probabilistic sampling will be used. All individuals identified through the collaborating organisations who meet the eligibility criteria will be invited to participate until the target sample size (N = 18) is reached.

##### Recruitment Strategies

In order to reach the sample size, we will partner with BAIGENE S.L. (Vitoria and Zamudio), the Judizmendi Civic Centre (Vitoria-Gasteiz City Council) and the Estadio Vital Fundazioa Foundation. Strategies include initial contact through their services and databases (having obtained the necessary authorisations), information material in their facilities and informative talks. The potential benefits of participation will be explained, and recruitment will be continuously monitored.

The recruitment scheme was structured into two stages and led by the principal investigator and the medical doctor in the research team in order to ensure rigorous selection:

##### Stage One: Information and Consent

In the initial meeting, the principal investigator or a trained member of the team will explain the study in detail (objectives, exercises), ensuring that the voluntary nature and expectations of the study are understood. Time will be allowed for questions. At the end of the meeting, stakeholders will sign an informed consent form.

##### Consent for Ancillary Studies with Biological Samples

A specific, optional informed consent will also be requested, in a separate section, for the storage and future use of the participants’ biological samples (saliva DNA) and associated (coded/anonymised) genetic data in ancillary studies on sarcopenia, muscle ageing, response to exercise or other future genetic analyses. Emphasis will be placed on the voluntary nature of participation and on ensuring that it does not interfere with their involvement in the main study.

##### Stage Two: Evaluation Criteria

After signing the consent form, participants will be assessed in order to confirm their eligibility and safety. This will include:

- Questionnaires and Clinical Information: Data on sociodemographic variables (age, sex), toxic habits (alcohol consumption), history of previous/current pathologies (reviewed by the team doctor to identify contraindications according to the exclusion criteria) and medication consumed will be collected. A Comprehensive Geriatric Assessment (CGA) will be conducted, evaluating key domains with brief, validated instruments. These will include: functional status for basic Activities of Daily Living (ADL) using the Katz Index of Independence in ADL; nutritional status using the Mini Nutritional Assessment—Short Form (MNA-SF); cognitive function using the Mini-Cog©; and depressive symptoms by means of the 5-item or 15-item Geriatric Depression Scale (GDS-5 or GDS-15). If available, parameters of interest (e.g., complete blood count, glucose, urea, creatinine with estimated GFR, serum electrolytes, total protein, albumin, C-reactive protein (CRP), Vitamin D, lipid profile) will be extracted from recent analyses.
- Anthropometric Measurements and Body Composition: Height will be recorded (in metres, to the nearest 0.01 m) using a stadiometer, with the participant barefoot and in light clothing. Weight (kg) and Body Mass Index (BMI) (kg/m^2^) will be obtained directly from the InBody 230 bioimpedance analyser (Biospace Co., Ltd., Seoul, Republic of Korea). The same device will be used to determine Skeletal Muscle Mass (SMM) and Appendicular Skeletal Muscle Mass (ASMM), following the manufacturer’s recommendations. The Appendicular Skeletal Muscle Mass Index (ASMMI), calculated as ASMM (kg)/height (m)^2^, will be used as a key component for the diagnosis of sarcopenia.

##### Diagnostic Tests for Sarcopenia (According to EWGSOP Criteria)

Specific diagnostic tests based on the EWGSOP criteria [2,3] will be performed to confirm the diagnosis of sarcopenia, a fundamental inclusion requirement:

- Running speed: This will be measured with a Newtest Powertimer system (Model 3000). Two series of 4 m will be performed, with the best result being chosen. Inclusion criteria: <0.8 m/s.
- Manual grip strength: This will be obtained with a Jamar dynamometer (Model 5030 J1) following a standardised protocol [3,77]. The dominant hand (or the contralateral hand if this is not possible) shall be used, with the participant seated and their elbow at 90°. The highest value of the three measurements (1-min interval) will be recorded. Inclusion criteria: males < 30 kg; females < 20 kg.
- Skeletal Muscle Mass: The acquisition of Skeletal Muscle Mass (ASMM or ASMMI using InBody 230) was described in the previous section (‘Anthropometric Measurements and Body Composition’). Inclusion criteria: men < 8.31 kg/m^2^ (ASMMI); women < 6.68 kg/m^2^ (ASMMI).

##### Inclusion Criteria

- Age 70 years or older.
- Diagnosis of sarcopenia according to EWGSOP criteria.

##### Exclusion Criteria

- Inability to make autonomous decisions (e.g., inability to give valid informed consent) or to understand and consistently follow exercise instructions, due to significant cognitive impairment (assessed by Mini-Cog© and clinical judgement that precludes safe and autonomous participation) or other conditions that severely impair comprehension or communication.
- Presence of active, unstable or severe medical conditions that, in the judgement of the study’s doctor, restrict effective participation or significantly increase the risk of injury associated with the programme, including, but not limited to:
  ○ Cardiovascular: Conditions leading to exclusion include uncontrolled arterial hypertension, defined as a resting Systolic Blood Pressure (SBP) > 180 mmHg or a resting Diastolic Blood Pressure (DBP) > 110 mmHg. Other exclusion criteria are a recent major cardiovascular event (within the last 6 months), such as unstable angina, myocardial infarction or cardiac surgery; decompensated congestive heart failure (New York Heart Association [NYHA] Class III–IV); uncontrolled, clinically significant ventricular arrhythmias; or symptomatic severe aortic stenosis.
  ○ Neuromuscular: Advanced or rapidly progressive degenerative neuromuscular diseases (e.g., Amyotrophic Lateral Sclerosis [ALS]) that preclude safe and effective exercise performance or could be exacerbated by IST.
  ○ Musculoskeletal: Active/severe multi-joint inflammatory arthritis, recent unconsolidated fractures in involved areas, major orthopaedic surgery < 6 months that contraindicates exertion, or severe chronic musculoskeletal pain significantly exacerbated by IST despite adjustments.
  ○ Respiratory: Severe Chronic Obstructive Pulmonary Disease (COPD), defined as stage III-IV according to the Global Initiative for Chronic Obstructive Lung Disease (GOLD) guidelines, with significant limiting symptoms, or severe, poorly controlled asthma.
  ○ Other severe or unstable medical conditions: Exclusion criteria also include advanced Chronic Kidney Disease (CKD), such as patients on dialysis or those with an estimated Glomerular Filtration Rate (GFR) < 30 mL/min/1.73 m^2^ without explicit approval from their nephrologist; uncontrolled diabetes mellitus with severe acute or chronic complications (e.g., untreated proliferative retinopathy, severe peripheral neuropathy with risk of ulceration); or any other pathology deemed an absolute contraindication to participation by the study’s doctor.
- Inability to commit to attending at least 90% of the scheduled training sessions during the 16 weeks of the study.

#### 2.1.2. IST Programme

The IST programme is meticulously structured to ensure a gradual progression and precise application of the stimulus. Each exercise will consist of a single continuous isometric contraction per muscle group, divided into three consecutive phases of specific intensity and equal duration, with no rest in between. The total duration of this contraction per exercise will be progressively increased over the 16 weeks of the protocol: 45 s (weeks 1–4), 60 s (weeks 5–8), 75 s (weeks 9–12) and 90 s (weeks 13–16).

The internal structure of each progressive isometric contraction is defined as follows:

- First phase: Isometric contraction to 20% of the MVIC determined in the pre-intervention assessment (see Section 2.2.2).
- Second phase: Consecutive increase of the intensity to 40% of the MVIC.
- Third phase: Maximal isometric effort (100% ME). The durations of these three phases are identical within a full contraction and are detailed in Table 2.

The weekly progression also includes an adjustment in the intensity of the submaximal phases: the strength requirement (% MVIC) in the first two phases will be increased by 2% every four weeks, as detailed in Table 2. A recovery time of 1 min will be established between the end of the series of one exercise and the start of the next. This progressive design aims to ensure that participants can gradually adapt to the effort required.

The CIEX SYSTEM Isometric Force Machine (Ciex Systems©, Durango, Spain) will be used to ensure correct execution and accurate monitoring of force. It will be equipped with Chronojump force sensors (Chronojump Boscosystems^®^, Barcelona, Spain) and Chronojump software (v2.5.1-8) for real-time visualisation of the force exerted. There will be progressive intensity changes between phases. The correct position will be established individually using a HALO© digital goniometer. While performing the exercise, participants will be instructed to maintain continuous breathing and avoid prolonged Valsalva manoeuvre, thus minimising acute tension responses [78,79]. Constant supervision will allow monitoring for any signs of discomfort.

The determination of the specific angle for each exercise and participant will be individualised. This will be monitored by the qualified personal trainer or principal investigator, guided by the following principles to ensure effectiveness, safety and comfort:

- Assessment of Active and Comfortable Range of Motion (ROM): The participant’s active and pain-free ROM for the main joint will be assessed at the beginning of the exercise, avoiding extreme ranges that generate discomfort, pain or instability, considering their ‘specific motor restrictions’.
- Selection of the Intermediate Angle within the Safe Range: An intermediate angle within the comfortable ROM will be selected that seeks to avoid joint locking or excessive passive tension, and allows the participant to generate a stable and effective Maximal Voluntary Isometric Contraction (MVIC). Although the peak torque angle will not be exhaustively sought, the practitioner will guide the participant to a position in the middle zone of their safe ROM where they subjectively report that they feel comfortable exerting force, favouring optimal actin and myosin overlap.
- Prioritisation of Safety and Tolerability: If in doubt, the angle that warrants the greatest perceived safety and comfort will be prioritised, even if it is not the theoretically ’optimal’ angle for maximal force production.
- Recording and Consistency: The individualised intermediate angle, once determined and accurately measured (HALO© digital goniometer), will be recorded and used consistently across all training sessions and MVIC assessments, ensuring standardised stimulus presentation. The aim of this process is to select a joint angle that represents a significant muscular challenge and allows for high fibre activation, within a zone that is biomechanically safe, personalised and well tolerated by each older adult.

**Figure 1:**
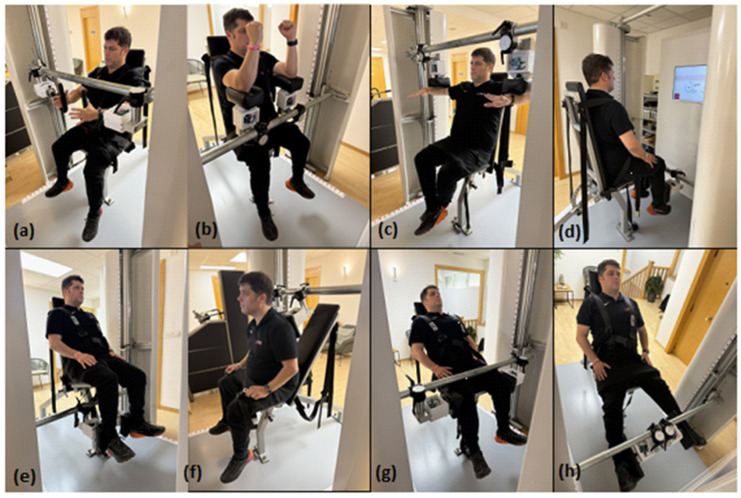
Starting and execution positions for the exercises of the Isometric Strength Training (IST) protocol: (**a**) Peck Deck, (**b**) Pull Over, (**c**) Lateral Shoulder Elevation, (**d**) Leg Extension, (**e**) Leg Curl, (**f**) Low Back, (**g**) Hip Abduction, (**h**) Hip Adduction.

The exercises to be performed are: peck deck, pull over, lateral shoulder raise, leg extension, leg curl, lumbar, hip abduction and hip adduction. A visual demonstration of these exercises is shown in Figure 1. The detailed standards for their application (positioning, main muscles, individualised determination of angles, instructions for the three contraction phases and monitoring points) are presented in Table 3.

##### Adherence to and Compliance with the Protocol

To promote adherence to the protocol and facilitate compliance, the following measures will be implemented:

- Each participant will attend individually, with a pre-established, adapted timetable.
- The sessions will be supervised and led by a qualified personal trainer and the principal investigator, who will provide ongoing support and motivation.
- If a participant misses two or more consecutive excused sessions, the intervention period may be extended by up to four additional weeks to allow time for recovery.

To comply with the protocol, participants must:

- Complete at least 90% of the scheduled sessions.
- Carry out all scheduled assessments within 20 weeks.
- Do not make significant changes in their daily lives that could interfere with the results or safety of the study. Specifically, participants will be instructed:
  ○ To maintain their usual dietary habits and not to start any special diets or significant nutritional supplementation without consulting the research team.
  ○ To refrain from starting new intense physical exercise programmes or substantially increasing their usual physical activity beyond the IST programme.
  ○ Continue with their usual medical care and prescribed treatments, as long as they do not directly interfere with the objectives and safety of the IST (as assessed by the research team). Any relevant new medication or treatment initiated will be recorded (see Section 2.1.3).

Non-compliance with these criteria will be recorded. The criteria for discontinuation or withdrawal from the study are detailed below:

##### Criteria for Discontinuation or Withdrawal of Participants During the Study

The IST progression protocol (Table 2) shall be applied in a standardised manner, with no individualised modifications to the progression. Persistent inability to follow the prescribed protocol, including unresolved mild discomfort, shall be managed by considering the following criteria for discontinuation or withdrawal:

1. Voluntary withdrawal by the participant: Participants may withdraw at any time and for any reason, as informed in the consent, without penalty or impact on the participant’s medical care.
2. Development or worsening of medical conditions: If, based on the medical judgement of the team or staff, a new condition or the worsening of a pre-existing condition contraindicates continuation with the IST or significantly increases the risk, participants may withdraw from the programme.
3. Adverse events (AEs): Occurrence of a Serious Adverse Event (SAE) or persistent/considerable AE related to participation, which, based on the medical judgement of the principal investigator or study doctor, makes it inadvisable to continue. All these occurrences will be recorded and evaluated according to the safety protocol.
4. Non-compliance with protocol:
  ○ Failure to complete a minimum of 90% of the sessions, even after considering the extension of the period.
  ○ Persistent inability or refusal to perform exercises according to instructions and proper technique, compromising safety or validity, despite supervision and correction.
  ○ Failure to complete scheduled assessments within 20 weeks.
5. Significant lifestyle changes or concomitant treatments: Initiation of unrelated intense exercise, drastic unsupervised dietary changes, or medical treatments that significantly interact with outcomes or safety, as assessed by the research team.
6. Failure to engage in follow-up sessions: If the participant becomes unresponsive and does not attend sessions/assessments, despite reasonable attempts at contact, discontinuation will be considered.

The main reason will be documented in all cases of discontinuation or withdrawal. Data collected up to that point will be securely retained and considered for inclusion in statistical analyses (e.g., intention-to-treat), as described in the ‘Statistical Analysis’ section. Participants who discontinue the intervention will be able to continue with scheduled assessments if they wish to do so, provided that this is feasible.

#### 2.1.3. Control Mechanisms for Field Protocol Standardisation

To ensure a standardised and rigorous implementation of the protocol and the quality of the data, the following control mechanisms will be put in place:

- Training of Research Staff: All staff directly involved in the intervention and evaluation (principal investigator, evaluation professionals, qualified personal trainer) will receive specific and detailed training on all study procedures. The ‘qualified personal trainer’ should have a degree in Physical Activity and Sports Science or Physiotherapy, with demonstrable experience in working with older adults and strength training. Training will cover: consistent application of inclusion/exclusion criteria; accurate technique for individualised determination of joint angles (HALO© digital goniometer); correct instruction, supervision and progression of IST exercises (CIEX System machine, Chronojump software with feedback); standardised administration of questionnaires; and accurate performance of all assessment tests. Recalibration sessions or periodic team meetings may be held to ensure consistency.
- Standardisation of the Intervention (IST Programme): The application of the IST will strictly follow the set progressive 16-week design (single set per muscle group, gradual increase of total stimulus duration and intensity of submaximal phases), as detailed in Table 2. The correct technical performance of the 8 specific exercises will be guided by the standards provided in Figure 1 and Table 3. The use of the CIEX SYSTEM machine with real-time feedback is essential in ensuring that target intensities are met. All sessions will be held individually and will be directly supervised by qualified personnel.
- Standardisation of Assessment Procedures and Minimisation of Bias: To minimise potential bias during assessments, standardised protocols for each test and data collection (detailed in Section 2.2) will be strictly followed, including specific recommendations (e.g., EWGSOP, InBody manufacturer). All assessment personnel shall be trained. Calibrated instruments and objective measures (CIEX System, InBody 230, Newtest Powertimer) shall be used. As far as possible, assessors will not have access to the previous results of participants to avoid expectation bias. If the analysis of the static posture photographs is conducted by a different individual, an effort will be made to blind them to the time point (pre/post) during the assessment. Although complete blinding of primary assessors at the time point is difficult in this design, strict adherence to protocols and the use of objective measures are the primary strategies to reduce observer bias.
- Monitoring of Adherence, Compliance and Concomitant Variables: Attendance to training sessions and assessments will be recorded in detail, and adherence to protocol criteria will be continuously monitored (e.g., ≥90% sessions, assessments on time). Changes in usual medication (by asking at assessments and training visits) will be recorded by referring to the baseline information in Section 2.1.1. Participants will be instructed to maintain their usual dietary and physical activity habits, not initiating new diets or intense exercise programmes; compliance will be monitored informally, and significant changes will be recorded. Standardised Case Report Forms (CRFs) will be used and made available for ethical consultation or audit if required.
- Strategies to Promote Retention and Completion of Follow-up: Individualised and flexible scheduling, close supervision with motivational support, regular communication, and the possibility of extending the intervention period to make up for excused absences will be used to promote retention. The importance of completing all phases will be explained. If a participant discontinues the IST, they will be encouraged to complete the post-intervention assessment (week 17) if feasible, to collect outcome data from as many participants as possible (providing intention-to-treat analysis). Data collected until withdrawal will be retained and managed according to the Section 2.3 (‘Statistical Analysis’).

These mechanisms aim to minimise variability in implementation and ensure data reliability.

#### 2.1.4. Data Management

The following plans will be implemented for the data management of the study:

- Standardised Case Report Forms (CRFs): Standardised (physical or digital) CRFs, designed specifically for this study, will be used for all variables. These CRFs will be available for ethical or audit consultation if necessary.
- Data Entry: Data from physical CRFs will be entered into a Microsoft Excel database. To ensure data quality, a second team member will thoroughly double-check all entered data against the original forms.
- Data Coding: Variables will be coded according to a pre-established data dictionary to ensure consistency. Personally identifiable data will be excluded from the analysis database, using anonymised participant identification codes instead.
- Data Quality: In addition to input verification, range and logical consistency checks will be performed to identify errors or outliers. Any inconsistencies will be reviewed and corrected in consultation with the original CRFs or measurement staff. Periodic data quality reviews shall be performed.
- Data Security and Storage: All data (physical forms and electronic database) will be stored securely. Electronic data will be stored in password-protected institutional computers, with access restricted to the principal investigator’s team. Regular encrypted backups will be performed and placed in a secure, locked external hard drive. Paper forms and informed consents will be stored under lock and key at Baigene S.L. In compliance with institutional regulations, good research practice, the EU General Data Protection Regulation (GDPR) and applicable Spanish legislation, all study data will be securely stored for a minimum of 15 years following the completion of the trial.

#### 2.1.5. Assignment of Intervention and Blinding

- Assignment of Intervention: This study employs a single-arm design where all participants receive the IST intervention. Therefore, no randomisation procedures, allocation concealment or implementation mechanisms will be applied to assign participants to different groups, as there is only one intervention group to which all eligible consenting participants will be assigned.
- Blinding:
  ○ Participants and Intervention Staff: Given the design and nature of the intervention (physical exercise), neither the participants nor the staff administering the IST (qualified trainer, principal investigator) will be blinded.
  ○ Outcome Evaluators and Data Analysts: Measures will be implemented to minimise evaluator bias (detailed in Section 2.1.3). Although complete blinding of lead assessors at the time point (pre/post) is difficult, attempts will be made to ensure that they do not access previous results when making subsequent measurements. For the analysis of static posture photographs, blinding of the analyst at the time of assessment will be attempted. Where possible, the primary statistical analysis shall be performed by a team member not involved in direct data collection; otherwise, the team will seek to analyse the primary data without knowledge of the temporal coding until preliminary analyses are completed, if feasible.
- Unblinding Procedure: Since there is no blinding of participants or staff to different treatment arms and the intervention is known, formal unblinding procedures are not applicable. In case of a serious adverse event, its relationship to the known IST intervention will be assessed according to safety protocols.

### 2.2. Assessment

The evaluation in this study will investigate the impact of the IST programme on the primary and secondary outcome variables detailed in Section 2.2.1. These variables encompass key indicators of sarcopenia, dynapenia, functional capacity, quality of life, and applicability and safety of the protocol in older adults. The outline of the pre- (week 0) and post-intervention (week 17, after 16 weeks of IST) assessments is described below. It is designed to obtain a comprehensive profile of each participant and measure the changes induced by the programme. Both phases will be conducted by a physical activity professional and the research group’s doctor.

#### 2.2.1. Study Outcome Variables (Outcomes)

The following variables will be assessed at pre- and post-intervention (week 0 and week 17, unless otherwise stated) to determine the efficacy and applicability of the IST, as distinguished from the eligibility measures (Section 2.1.1):

A. Primary Outcome Variables (Assessment of reversal of dynapenia and sarcopenia):

1. Change in MVIC Knee Extensors:
  ○ Specific measurement variable: MVIC of the knee extensors, measured in Newtons (N) with the CIEX SYSTEM machine.
  ○ Analysis metric: Absolute change (MVIC_week17_–MVIC_week0_)
  ○ Method of aggregation: Mean and Standard Deviation (SD) of the change. The percentage change will also be calculated.
  ○ Time point(s): Baseline (week 0) and post-intervention (week 17) assessments.
  ○ Clinical Relevance: Knee extensor strength is key to functionality and independence in older adults; its improvement indicates reversal of dynapenia.
2. Change at ASMMI:
  ○ Specific measurement variable: ASMMI (ASMM in kg/height^2^ in m^2^), measured by bioimpedance (InBody 230).
  ○ Analysis metric: Absolute change (ASMMI_week17_–ASMMI_week0_)
  ○ Method of aggregation: Mean and SD of change.
  ○ Time point(s): Baseline (week 0) and post-intervention (week 17) assessments.
  ○ Clinical Relevance: Fundamental diagnostic criterion for sarcopenia (EWGSOP2); an increase indicates improvement in the amount of muscle, essential for metabolic health and reduced frailty.

B. Secondary Outcome Variables

1. Other Indicators of Sarcopenia and Dynapenia:
  ○ Handgrip Strength: Specific measurement variable: Maximal handgrip strength, measured in kilograms (kg) (Jamar Dynamometer). Analysis metric: Absolute change from baseline to week 17. Method of aggregation: Mean and SD. Time point(s): Baseline (week 0) and post-intervention (week 17). Clinical Relevance: A component of the sarcopenia diagnosis and a predictor of functionality and mortality.
  ○ Gait Speed: Specific measurement variable: Usual gait speed over a 4 m course, measured in meters/second (m/s) (Newtest Powertimer). Analysis metric: Absolute change from baseline to week 17. Method of aggregation: Mean and SD. Time point(s): Baseline (week 0) and post-intervention (week 17). Clinical Relevance: A measure of physical performance, a component of the sarcopenia diagnosis, and a predictor of adverse outcomes.
  ○ MVIC of other muscle groups: Specific measurement variable: MVIC for the peck deck, pull over, lateral shoulder elevation, leg curl, low back, hip abduction and hip adduction, measured in Newtons (N) (CIEX SYSTEM). Analysis metric: Absolute change from baseline to week 17. Method of aggregation: Mean and SD of the change per exercise. Time point(s): Baseline (week 0) and post-intervention (week 17). Clinical Relevance: Assesses the training response across different body regions.
  ○ Sarcopenia Status: Specific measurement variable: Diagnostic status of sarcopenia according to EWGSOP2 criteria (based on muscle strength, muscle mass, and physical performance). Analysis metric: Change in the proportion of participants meeting the diagnostic criteria from baseline to week 17. Method of aggregation: Frequencies and percentages. Time point(s): Baseline (week 0) and post-intervention (week 17). Clinical Relevance: Quantifies the overall impact of the intervention on the diagnostic status of the condition.
2. Health-Related Quality of Life (HRQoL):
  ○ Specific measurement variable: The index score derived from the EQ-5D-5L descriptive system and the Visual Analogue Scale (VAS) score of the same questionnaire. Analysis metric: absolute change from baseline to week 17 for both the index score and the VAS score. Method of aggregation: Mean and SD of the changes. Time point(s): Baseline (week 0) and post-intervention (week 17). Clinical Relevance: Measures the overall self-perceived health and the impact of the intervention on multiple dimensions of participant well-being.
3. Applicability of the IST Protocol:
  ○ Perceived Safety: Specific measurement variable: Score on a 5-point Likert scale for perceived safety. Analysis metric: Mean score post-intervention. Method of aggregation: Mean and SD. Time point(s): Post-intervention (week 17). Relevance: Assesses the participant’s subjective perception of safety, providing data to test hypothesis H1.
  ○ Acceptance/Satisfaction: Specific measurement variable: (1) Adherence rate (% of completed sessions); (2) Score on a 5-point Likert scale for overall satisfaction. Analysis metric: Percentage for adherence; Mean score post-intervention for satisfaction. Method of aggregation: Percentage for adherence; Mean and SD for satisfaction score. Time point(s): Adherence monitored continuously during the intervention (weeks 1–16); Satisfaction assessed post-intervention (week 17). Relevance: Measures the feasibility and appeal of the intervention to participants, providing data to test hypothesis H2.
  ○ Perceived Difficulty: Specific measurement variable: Score on a 5-point Likert scale for perceived difficulty. Analysis metric: Mean score post-intervention. Method of aggregation: Mean and SD. Time point(s): Post-intervention (week 17). Relevance: Evaluates the perception of technical simplicity, providing data to test hypothesis H3.
  ○ Adverse events: Specific measurement variable: Incidence, type, severity and causality (relationship to IST). Analysis metric: Frequencies and descriptive summary. Method of aggregation: Counts and percentages. Time point(s): Monitored continuously during the intervention period (weeks 1–16). Relevance: Assesses the objective safety profile of the protocol, providing data to test hypothesis H1.

* Relevance (common to the previous 4): To evaluate the feasibility, safety, acceptability and perceived difficulty of the IST protocol from the participants’ perspective.

4. Static Posture Parameters:
  ○ Specific measurement variable: Key angular and alignment parameters of static posture (e.g., craniovertebral angle, shoulder symmetry, pelvic tilt), assessed from photographs using Kinovea^®^ software. Analysis metric: Absolute change from baseline to week 17, measured in degrees or centimetres. Method of aggregation: Mean and SD of the change for each parameter. Time point(s): Baseline (week 0) and post-intervention (week 17). Clinical relevance: Improvements in postural alignment may be associated with reduced musculoskeletal pain and better overall physical function.
5. Physiological Variables at Rest:
  ○ Specific measurement variable: Resting systolic blood pressure (SBP) and diastolic blood pressure (DBP) in mmHg, heart rate (HR) in bpm, and peripheral oxygen saturation (SpO2) in %. Analysis metric: Absolute change from baseline to week 17. Method of aggregation: Mean and SD of the changes for each variable. Time point(s): Baseline (week 0) and post-intervention (week 17). Clinical relevance: These variables are monitored primarily for participant safety. Additionally, based on evidence that isometric training may positively influence resting blood pressure (BP) over the medium term [79,80], changes in SBP and DBP will be analysed as a secondary outcome of clinical interest.

C. Exploratory Outcome Variables

- Genetic Association: Analysis of the association between selected genetic polymorphisms and the magnitude of the IST response in primary and secondary outcomes. This will be explored using regression models or genotype group comparisons.
- Other Descriptive Variables: Baseline data from the CGA and laboratory analyses will be used to characterise the sample and to explore other potential factors associated with the training response.

#### 2.2.2. Pre-Intervention Assessment

One week before starting the 16-week IST, a comprehensive baseline assessment will be performed on the selected subjects to collect baseline data. This assessment, conducted by a physical activity professional and the research group doctor, will allow for detailed monitoring and accurate assessment of the effects of the programme, including the following variables:

- Physiological Variables: BP and heart rate HR (Omron M3 Comfort^®^ blood pressure monitor, HEM-7134-E, Omron Healthcare Co., Kyoto, Japan) and O2 saturation (OXYM4000 pulse oximeter, Quirumed S.L.U., Valencia, Spain) will be measured. Three resting measurements will be taken per participant, using the mean as the final data.
- Functional Capacity:
  ○ MVIC: The MVIC of each muscle group of the IST programme will be assessed at individualised intermediate angles (determined according to anatomical structure and motor restrictions, recorded with a HALO© digital goniometer). The CIEX SYSTEM machine (Ciex Systems©, Durango, Spain) with sensors and Chronojump software (Chronojump Boscosystems^®^, Barcelona, Spain) will be used for instantaneous measurement and visualisation of strength, tools also used in all IST sessions and for monitoring purposes.
  ○ Static Posture Assessment: Participants will be photographed in anatomical position (frontal and sagittal planes) with a Casio Exilim Pro EX-F1 camera (configuration: level tripod, lens at umbilical height, at 4 m, optical zoom, aperture priority, focal length 50 mm, f5.6, ISO 200) [81]. An analysis of documented validity and reliability for postural analysis will be performed with Kinovea^®^ software (v.0.9.x+) [82,83]. Adhesive markers or direct image identification may be used for key anatomical points [84] (sagittal: tragus, C7, acromion, greater trochanter, lateral femoral epicondyle, peroneal malleolus; frontal: acromions, anterior superior iliac spine (ASIS), posterior superior iliac spine (PSIS), patellar centre, tibial malleoli). Postural parameters will be calculated from these points (sagittal: head-neck alignment (HNA) [82], shoulder alignment, estimated spinal curvatures, pelvic tilt [84], knee alignment; frontal: head tilt/symmetry, shoulder height, pelvic symmetry [81], lower limb alignment [82]). Angles (degrees) and distances (cm/mm, after calibration if size reference is used) shall be recorded. Pre-post change will be an outcome of interest. Image analysis shall be performed, if possible, by a blinded assessor (see Section 2.1.3) [83].
- HRQoL: The EuroQol EQ-5D-5L questionnaire (5 items, 5 dimensions, 5 response levels) will be used [85,86]. This instrument includes two parts: a descriptive system comprising five dimensions (mobility, self-care, usual activities, pain/discomfort, anxiety/depression), each with five response levels, and a VAS where participants rate their overall health.
- Applicability of the IST Protocol (scales administered post-intervention):
  ○ Safety (H1): Assessed by: (1) Analysis of Adverse Events (AEs) (incidence, type, severity, relationship to IST) over 16 weeks (procedures detailed in Section 2.4.3); ‘low risk’ if the incidence of IST injuries is minimal. (2) Perceived Safety: 5-point Likert scale (1 = extremely unsafe, 5 = extremely safe) post-programme, with face validity and based on similar approaches [87]; a mean score ≥ 4 will be expected.
  ○ Acceptability (H2): Assessed by compliance rates (≥90% sessions) and overall satisfaction (5-point Likert scale: 1 = extremely dissatisfied, 5 = extremely satisfied) post-programme, using validated measures [88]; a mean satisfaction score ≥ 4 will be expected.
  ○ Difficulty (H3): Assessed with a 5-point Likert scale on perceived challenge (1 = not challenging at all, 5 = extremely challenging) post-programme, validated approach [89]; a mean score ≤ 2 will be expected.
- Genetic Analysis: Saliva samples will be collected using buccal swabs (4N6FLOQSwab, Life Technologies, Waltham, MA, USA). DNA will be extracted from these samples (QIAmp DNA Mini kit, Qiagen, Venlo, The Netherlands) and subsequently quantified (Qubit, Life Technologies). Genetic polymorphisms, specifically Single Nucleotide Polymorphisms (SNPs) and Insertions/Deletions (INDELs), will be analysed in genes associated with the response to strength training, hypertrophy, and strength gains [63,64,65,66,67,68,69,71,72,73]. Specifically, genes linked to the following will be analysed:
  ○ Muscle fibre typology: such as ACTN3 (R variant associated with a higher proportion of type II fibres, crucial for strength/power and affected by sarcopenia/dynapenia in ageing) [63,64].
  ○ Response to training: including ACE [63,65,66], NRF2, CKMM, MCT1, AMPD1, and the co-activators PPARGC1A and PPARGC1B (involved in mitochondrial biogenesis and exercise adaptation).
  ○ Muscle fibre damage and regeneration: genes such as IGF2, MLCK, CASP [72], MMP3 [72], TNC and GDF5 [73], which influence the response to exercise microtrauma and its repair. Moderate exercise-induced muscle damage (EIMD), potentially triggered by IST at long angles, is considered to contribute to remodelling and adaptation.
  ○ Vascularisation: NOS3 and VEGFA [67,69], essential for oxygen/nutrient supply, muscle growth and recovery, optimising metabolic stress and hypertrophic response.
  ○ Inflammation: TNF-α, CRP [60], IL-6 [60] and IL6-R, which are relevant because inflammation contributes to sarcopenia and modulates the anabolic response [70].
  ○ Oxidative damage response capacity: SOD2 [71], CAT and GPX, crucial for muscle recovery and adaptation to training stress.
  ○ Fat and carbohydrate metabolism (nutrigenomic factors): Polymorphisms in FABP2, ADRB2, ADRB3, FAT/CD36, UCP2, GCKR, ADCY5 and GIPR, which may influence nutrient uptake and hypertrophic response [74,75,76].

Genetic analysis will be conducted using the Biomark HD system (Standard BioTools, formerly Fluidigm; South San Francisco, CA, USA) in the laboratory facilities of Baigene S.L. (Álava Technology Park, Spain).

#### 2.2.3. Post-Intervention Evaluation

A final assessment will be performed one week after completion of the 16-week IST programme (week 17). All baseline measurements (physiological variables, functional capacity including MVIC and posture, quality of life by EQ-5D-5L, and the components of the sarcopenia diagnosis) will be repeated to assess any changes induced by the training, with the exception of the genetic analysis, which is only performed at baseline. Additionally, applicability questionnaires (safety, acceptance and perceived difficulty using Likert scales) [87] will be administered to assess the IST protocol.

### 2.3. Statistical Analysis

This study will employ a single-group design with pre- and post-intervention measurements. The analysis will follow an intention-to-treat (ITT) and, complementarily, per-protocol (PP) approach for participants with ≥90% session attendance. Missing data will be minimised through rigorous follow-up and promotion of retention (Section 2.1.3). The pattern and amount of missing data will be analysed; if <5% and MCAR (Missing Completely At Random), complete case analysis will be chosen for certain variables. For the main ITT analysis with larger missing data or with a specific pattern, multiple imputation techniques or mixed models (under the MAR—Missing At Random assumption) will be considered.

#### 2.3.1. Sample Size Calculation

The sample size was determined after an internal pilot study (N = 50) conducted by the research team (Baigene), using a similar training protocol (CIEX SYSTEM machine) but without visual strength feedback. It was carried out in a population aged ≥65 years with and without sarcopenia/dynapenia and had a duration of 10 weeks. The primary variable for calculation was quadriceps MVIC (N). Pilot results (mean ± SD, Pre vs. Post):

- Left quadriceps: 224.78 ± 64.49 N vs. 296.55 ± 89.38 N; Cohen’s d (paired samples) = 1.22.
- Right quadriceps: 263.53 ± 84.66 N vs. 306.24 ± 106.74 N; Cohen’s d (paired samples) = 0.51. Effect sizes were calculated with the mean and SD of the pre-post differences. Given the expected greater improvement with feedback in this protocol, the more specific population (≥70 years with sarcopenia/dynapenia) and the longer duration of the current intervention, a (conservative) d = 0.80 was adopted for the calculation. With G*Power v3.1.9.7, for a related samples *t*-test, detecting d = 0.80 with 80% power and α = 0.05 (bilateral) requires N = 15. Adjusting for 15% losses, 18 participants will be recruited, an adequate number for this pilot exploratory study.

#### 2.3.2. Analysis Strategy

Primary and secondary quantitative variables will be compared pre-post with Student’s *t*-tests for related samples, after testing for normality (Shapiro-Wilk). If the data are not normally distributed or contain outliers, non-parametric tests (e.g., the Wilcoxon signed-rank test) will be employed. Effect sizes (Cohen’s d) and confidence intervals will be calculated. Multivariate analyses will include a multiple linear regression to explore the influence of genetic and environmental variables on strength and muscle mass improvement, with Bonferroni correction for genetic analyses. Qualitative variables (e.g., sarcopenia diagnosis) will be analysed by using McNemar tests. Applicability variables (Likert scales, adverse events) will be analysed with descriptive statistics and *t*-tests or Wilcoxon tests for pre-post comparisons on Likert scales. Additionally, exploratory subgroup analyses will be performed to assess whether the response to IST on primary variables differs according to sex, age ranges (70–79 vs. ≥80 years) and baseline severity of sarcopenia/dynapenia. Adjustment for baseline covariates will be considered in regression models. The software will be IBM SPSS v29.0 and R v4.3.1.

For the assessment of the secondary hypotheses concerning the applicability (Section 2.2.2) of the IST protocol, specific analyses will be carried out:

Hypothesis H1 (low injury risk): This will be assessed through two approaches. First, Adverse Events (AEs) will be recorded (including their type, severity, and causality), and ‘low risk’ will be inferred if the incidence of IST-related injuries is minimal and comparable to or lower than rates reported in the literature for strength training in older adults [1,33,34,35]. Second, Perceived Safety will be measured post-intervention using a 5-point Likert scale; the approach for this subjective scale is based on similar methods used in previous research [87]. The hypothesis will be supported by a mean score of ≥4.

Hypothesis H2 (high acceptance): This will be assessed by: (1) compliance rate, with a success threshold of ≥90% of completed sessions, and (2) a satisfaction score from a 5-point Likert scale administered post-intervention, using a validated measurement approach [88]. The hypothesis will be supported by a mean satisfaction score of ≥4.

Hypothesis H3 (low perceived difficulty): This will be assessed using a 5-point Likert scale for perceived technical difficulty post-intervention, based on validated methods [89]. The hypothesis will be supported by a mean score of ≤2.

Hypothesis H4 (Genetic influence). The association between polymorphisms (muscle typology [63,64], inflammation [70,71], tissue regeneration [72,73], energy metabolism [74,75,76]) and improvements in mass/strength (IST) will be analysed by multiple linear regression, adjusting for covariates and with the Bonferroni correction test.

For the Likert-based outcomes (H1–H3), the magnitude of change will also be assessed by calculating Cohen’s d.

### 2.4. Monitoring of the Trial

#### 2.4.1. Data Monitoring Committee (DMC)

Given the anticipated low risk of the IST intervention, the non-pharmacological nature of the trial, its single-arm design and its scale (N = 18), a formal, independent DMC has not been constituted. Monitoring of participant safety and study integrity will be the ongoing responsibility of the principal investigator (Iker López) and the research team, including their thesis supervisors (Juan Mielgo-Ayuso and Arkaitz Castañeda-Babarro). Regular team meetings will be held to review progress, adherence and any adverse events reported.

#### 2.4.2. Interim Analyses and Trial Stopping Guidelines

No formal interim analyses of efficacy or futility are planned that would lead to early stopping of the trial. The study will continue until completion of the 17-week follow-up of all participants, unless unforeseen safety concerns warrant an overall reconsideration of the protocol by the principal investigator and the ethics committee, who would make a decision on early termination for safety reasons.

#### 2.4.3. Collection, Assessment, Reporting and Management of Adverse Events (AEs)

Active safety monitoring will be performed throughout the study.

- Collection: In each training session, the trainer will actively ask about unusual complaints or symptoms (requested AEs). Participants will be instructed to spontaneously report any AEs between sessions to the principal investigator or trainer. All AEs will be systematically recorded.
- Assessment: The description, dates, intensity (mild, moderate, severe), seriousness (if Serious Adverse Event—SAE), measures taken and causality (relationship to SAE) of each AE will be documented by the principal investigator and the study doctor. A SAE will be defined as one that results in death, is life-threatening, requires hospitalisation/prolongation of life, results in significant disability/incapacity, or is a congenital anomaly.
- Reporting: All AEs will be recorded in each participant’s CRF. AEs will be reported to the Research Ethics Committee of the University of Deusto according to its regulations and deadlines.
- Management: Care will be provided in the event of an AE. If the AE is mild and related to the intervention, a temporary break may be considered (although progression is standardised). If the AE is moderate, severe, or considered a SAE, or if deemed necessary for participant safety by the principal investigator or study doctor, discontinuation of the participant’s involvement will be considered (as detailed in Section 2.1.2). Any participant who suffers harm as a result of trial participation will receive appropriate medical treatment.

#### 2.4.4. Audit of Trial Conduct

Given the exploratory, low-risk and doctoral thesis nature of this single-arm study, no formal, independent external audits are planned. Monitoring of compliance with the protocol and good research practice will be the ongoing responsibility of the principal investigator, under the supervision of their thesis supervisors and in collaboration with the research team. The Research Ethics Committee of the University of Deusto may carry out such reviews or request such information as it deems appropriate.

## 3. Discussion

The present 16-week IST protocol aims to evaluate its effect on sarcopenia, dynapenia, quality of life and applicability in adults aged 70 years and above, while also analysing the influence of genetic and environmental variables on the variability of the response.

Programmed strength training is the best strategy to combat sarcopenia and dynapenia [1,5,16,17]. To stimulate type II fibres, protocols have evolved towards dynamic explosive actions with light loads and lifting high loads to muscle failure [1]. However, these TRT methodologies require high technical proficiency and effort, making them difficult to apply in older adults due to physical and psychological limitations. The need to execute complex dynamic movements can be intimidating or painful for older adults with comorbidities (osteoarthritis, chronic pain, reduced mobility) and psychological barriers (fear of falling, low self-efficacy, perception of gymnasiums). Lack of mobility and difficulty maintaining technique with high loads or ballistic movements increase the risk of injury [1], underlining the need for more suitable alternatives.

IST, due to its biomechanical characteristics, could be an effective and safe option. It is not technically difficult, does not require movement and allows angles to be adjusted to individual needs, overcoming barriers such as apprehension of complex movements or pain induced by them. This facilitates concentration of effort, favouring a more accessible activation of high-threshold fibres. Static and controlled maximal effort can be safer and psychologically more approachable.

When designing an IST programme, the intensity and duration of contractions are key variables. The literature indicates that longer duration contractions (e.g., 30 s) may induce greater hypertrophy than multiple short contractions with equal time under tension [51]. Likewise, IST with high resistance or maximal effort seems particularly effective for strength [52]. Our protocol incorporates these elements: a maximal effort phase (100% ME) and a progressively extended total contraction duration (45–90 s). Additionally, the gradual progression of intensity within each set (20% MVIC, 40% MVIC, 100% ME) is based on practical and physiological considerations for older adults. Unlike protocols that reach maximum intensity directly, our gradual approach seeks to minimise perceived aggressiveness and risk. It is hypothesised that incipient fatigue prior to maximal effort may modulate the absolute force exerted, acting as self-protection without compromising the stimulation of type II fibres. This structure aligns with Henneman’s size principle [32], ensuring an orderly recruitment of motor units (Type I to Type II). The implementation of a single progressive set per muscle group also responds to criteria of efficiency, tolerability and the nature of the maximal isometric stimulus. A single set of considerable duration (45–90 s) culminating in 100% ME is designed to induce high muscle activation and fatigue, potentially sufficient for the desired adaptations, which is consistent with findings suggesting optimal muscle activation at high effort or task failure without the need for multiple sets [31]. For older adults, a single-set protocol is more time-efficient, may reduce systemic fatigue, and improve adherence and manageability, optimising safety, tolerability and metabolic effect.

The selection of intermediate joint angles is another key methodological consideration. While IST at LML and MML may generate greater improvements in strength and mass [44,45,49,50] and maximise mechanical strain, it may also be associated with greater EIMD [50,90]. For our population (>70 years with sarcopenia), with potentially diminished recovery capacity and lower tolerance to pain or extreme joint stress, we opted for intermediate angles. This choice seeks to optimise active force generation (length-tension ratio) while minimising excessive joint/tendon stress and significant EIMD, prioritising safety and tolerability.

The benefits of this IST protocol on strength and muscle mass are expected to be due to combined neuromuscular mechanisms. Neurally, 100% ME seeks to maximise the activation and firing rate of high-threshold motor units, following Henneman’s principle [32]. Muscularly, the mechanical strain of long, high-intensity isometric contractions is a potent stimulus for protein synthesis and hypertrophy [42,43,44,45]. Gradual progression of intensity and duration will facilitate these adaptations. Metabolic stress from vascular occlusion may contribute to this, and the choice of intermediate angles, in addition to safety, aims to optimise the generation of active tension by favouring a greater number of actin-myosin cross-bridges.

Several studies have shown that IST can significantly increase muscle strength and hypertrophy [42,43,44,45], positioning it as an effective tool, possibly more so than TRT, to combat sarcopenia and dynapenia in the elderly.

Beyond the technical complexity, TRT with explosive execution and high loads increases the risk of injury [33,34,35]. In contrast, IST is used in rehabilitation and special populations [36,37,38,39] because it allows safe strength work with minimal or no pain in specific ranges [36,37]. This is a practical advantage for older people with joint pain, as strengthening without exacerbating pain improves viability and can have acute analgesic effects [57,58], improving quality of life. The lower likelihood of severe DOMS and lower perceived technical difficulty may also favour acceptance of and adherence to IST.

One consideration in high-intensity IST is an acute BP response. Although maximal isometric contractions may transiently elevate BP [80,91], our protocol seeks to mitigate this risk by including participants with controlled hypertension, gradual progression of intensity, instruction for continuous breathing (avoiding prolonged Valsalva) [78,79], and constant monitoring.

Therefore, IST could be considered a safer and more effective alternative to TRT in older adults to improve their quality of life.

After the intervention has ended, it is important to anticipate difficulties and consequences. Long-term maintenance of benefits and adherence outside a supervised setting are key challenges, requiring transition strategies to sustainable practices. Transferring this protocol to community or home-based settings through the use of specialised equipment (CIEX SYSTEM) will also pose some difficulties, requiring more accessible adaptations to be explored. The main expected post-intervention consequence is progressive detraining if exercise is not maintained, underlining the need for continuous physical exercise. Additionally, psychological effects may arise from the termination of study support.

On the other hand, while the triggers for sarcopenia and dynapenia, and their variability, have not been fully identified, they can include inflammation, obesity, hormonal changes, inactivity and oxidative stress [92]. The inclusion of genetic polymorphism analysis could provide important information on the physiological causes and variability in recovery after IST and would be a valuable tool for interpreting the observed improvements.

## 4. Limitations and Strengths of the Study

The results will depend on the comparison between initial and final assessments, and it is crucial that the subjects maintain similar physical and psychological conditions. Working with a vulnerable population may limit the collection of completely objective data despite their actual evolution.

A significant methodological limitation is the absence of a parallel control group. The current single-arm design was chosen for this initial phase to assess the feasibility and preliminary effects of the IST protocol before proceeding to a larger-scale Randomised Controlled Trial (RCT). This feature compromises internal validity, making it difficult to attribute changes to the IST alone with any degree of certainty; factors such as natural progression of the condition, Hawthorne effects or uncontrolled variables may play a role. The inclusion of a control group, preferably through an RCT, as a strategy for future research would strengthen the ability to establish a direct causal relationship and improve internal validity.

Another limitation that may affect internal validity is the exhaustive control and quantification of lifestyle variables (diet, spontaneous physical activity) and medication changes unrelated to the intervention. Although general data are collected (in comprehensive geriatric assessment and for genetic analysis) and participants are instructed to maintain their habits (Section 2.1.3), the absence of detailed baseline assessment and continuous monitoring of these factors limits the ability to completely rule out their role as confounders. In order to partially mitigate this, an analysis of covariance (ANCOVA) could be employed to adjust the results for certain variables, provided they are reliably measured and significant changes are recorded. Future studies would benefit from more rigorous experimental control or more detailed measurement of these confounders.

Additionally, although multiple measures will be implemented to standardise assessments and minimise observer bias (staff training, adherence to detailed protocols, use of objective and calibrated instruments, and blinding of the posture imaging analyst, detailed in Section 2.1.3), complete blinding of the lead assessors to pre- or post-intervention status is difficult to guarantee in a single-arm design with the same staff involved in follow-up. This potential lack of complete blinding could be considered a limitation, mitigated by the stringency and objectivity of the measurement protocols.

Despite these limitations, the present study aims to generate initial evidence on the feasibility, safety and potential effects of a specific IST protocol in older adults with sarcopenia and dynapenia, as described in the objectives. On the other hand, among the strengths of the study is that, to the best of our knowledge and following a review of the literature, there is a notable paucity of published research specifically addressing maximal IST in the elderly with the methodological approach proposed here. This makes its results a possible novelty with worldwide relevance, given the growing importance of strength training among this population. In addition, the inclusion of genetic variables could provide key information to understand the variability in the improvement results, analyzing the influence of these variables on the response to IST.

## 5. Conclusions

IST is emerging as an exercise modality with considerable potential to be more efficient and safer than TRT in older adults. This is mainly due to its lower technical complexity, adaptability to individual mobility, reduced risk of injury (as it does not involve movement or speed), and its ability to effectively stimulate high-threshold muscle fibres, highlighting its relevance in the prevention and treatment of sarcopenia and dynapenia.

If the results confirm the efficacy and applicability of the proposed IST protocol, the clinical and practical applications would be significant, including its inclusion into geriatric rehabilitation, exercise prescription in primary care and community programmes. A validated IST protocol could improve functional independence, reduce the risk of falls, optimise the management of sarcopenia and dynapenia, and improve quality of life. In the longer term, information from genetic analysis could provide the basis for more personalised exercise strategies.

This study will also open up future avenues of research. Randomised Controlled Trials (RCTs) comparing this IST protocol with other interventions (IST, different modalities of TRT, control groups) will be essential. Future research should explore the long-term effects of IST, sustained post-study adherence, and strategies to adapt the protocol to less specialised equipment or home settings to maximise accessibility. Further investigation of the physiological mechanisms of this IST protocol and continued research on response-modulating genetic profiles will be areas of interest.

The results of this protocol will be presented at scientific conferences and published in peer-reviewed journals in geriatrics, sports medicine, exercise physiology and rehabilitation, aiming for open access. The results are expected to be published after completion of data collection and analysis. The study protocol is also intended for publication.

Ultimately, this protocol seeks not only to evaluate a promising intervention but also to lay the groundwork for future research and practical applications that contribute to healthier and more active ageing.

## 6. Protocol Administrative Information and Trial Governance

### 6.1. Trial Identification and Protocol

This protocol (Version 2.0, 29 May 2025) describes a trial entitled ‘Protocol for a Trial to Evaluate the Efficacy and Applicability of Isometric Strength Training in Older Adults with Sarcopenia and Dynapenia’. It will be registered in a public clinical trials registry (e.g., REec or ClinicalTrials.gov) prior to the start of recruitment, providing all elements of the World Health Organization (WHO) Trial Registration Dataset. The identifier will be included in future communications.

### 6.2. Funding and Support

This study has not received specific funding other than the ordinary resources of the participating institutions. The first author holds the patent to the specialised equipment (CIEX SYSTEM Isometric Force Machine). The facilities will be provided by the Judizmendi Civic Centre of the Vitoria-Gasteiz City Council and the Estadio Vital Fundazioa Foundation. The genetic analyses will be carried out by the company Baigene S.L. The Universities of Burgos and Deusto, and Kirolene, provide institutional, academic and human support.

### 6.3. Roles and Responsibilities

The names and affiliations of the contributors to the protocol and specific roles are detailed on the title page. Their main roles in study conception, design, methodology development and manuscript writing are specified below:

- Iker López: Conception of the study, design of the intervention, principal investigator, and writing the original draft.
- Juan Mielgo-Ayuso: Thesis direction, substantial contribution to the conception and design of the study, methodological design, supervision of protocol development and critical review of the manuscript.
- Juan Ramón Fernández-López: Methodological design, critical review.
- Jose M. Aznar: Contribution to the design of genetic analysis, critical review.
- Arkaitz Castañeda-Babarro: Co-direction of the thesis, statistical advice, contribution to the methodological design and critical revision of the manuscript.

The sponsor of the trial is the University of Burgos, under which the principal investigator’s PhD thesis is being completed. The principal investigator’s contact details (for enquiries about the study) are: Iker López, Department of Health Sciences, Faculty of Health Sciences, University of Burgos, ilt0002@alu.ubu.es. Institutional contact of the sponsor: Doctoral School of the University of Burgos (ED-UBU), Hospital del Rey, s/n, 09001 Burgos (Spain), info@ubu.es, +34 947 25 87 00. The University of Burgos provides the academic framework and supervision of the thesis, without direct involvement in data collection, day-to-day management, specific analysis or the final publication decision, beyond the standard academic evaluation. Baigene S.L. is responsible for the genetic analysis and provides expert advice in this area, integrating its results into the overall analysis by the research team. The organisations providing facilities and logistical support have no role in the design, analysis or publication of the paper. Given the absence of specific external funding that imposes conditions, the principal investigator and his team will maintain final authority over all phases of the study and publications, ensuring scientific independence. Overall coordination and supervision are the responsibility of the principal investigator, with the support of his PhD supervisors (Juan Mielgo-Ayuso and Arkaitz Castañeda-Babarro) and co-investigators. The study is coordinated by the Department of Health Sciences of the University of Burgos. No formal steering or event adjudication committees have been set up, given the nature of the study. Data management (detailed in Section 2.1.4) and data quality will be the responsibility of the principal investigator, with statistical advice from Arkaitz Castañeda-Babarro.

#### Confidentiality and Data Protection

The confidentiality of participants’ personal information will be protected before, during and after the study, in compliance with the GDPR (EU) 2016/679 and applicable Spanish legislation. Data will be collected with coded CRFs; each participant will be assigned a unique code, and the linkage list will be stored separately and securely with restricted access. Biological samples and their genetic results will also be encrypted. Access to personally identifiable data will be restricted to the research team involved in the follow-up. Any data shared or published will be aggregated and anonymised. Data storage and maintenance are described in Section 2.1.4. After the minimum required retention period (specified in Section 2.1.4), identifiable data will be securely destroyed, unless explicit consent is given for prolonged retention. Participants will be informed of these measures in the consent process.

## 7. Ethical and Regulatory Considerations

### 7.1. Research Ethics Approval

This protocol has been submitted to the Research Ethics Committee (REC) of the University of Deusto for evaluation. The REC is expected to provide its formal approval before initiating any study procedure with participants.

### 7.2. Informed Consent Process

The detailed process of informing potential participants, obtaining their written informed consent and identifying the responsible research team members is described in Section 2.1.1 (‘Step 1: Information and Consent’). Specific and optional consent will be sought for the storage and future use of biological samples and genetic data in ancillary studies, as also detailed in Section 2.1.1. Each potential participant will be provided with a detailed Information Sheet and an Informed Consent Form, with sufficient time for questions and decision-making. A model of the Informed Consent Form is the Supplementary Material.

### 7.3. Biological Samples: Collection, Analysis, Storage and Future Use:

Collection, DNA extraction and genetic analysis will be performed according to Section 2.2.2 (‘Genetic Analysis’). Samples and extracted DNA not fully utilised, and for which consent for future use has been obtained (Section 7.2), will be stored in coded form in the laboratory of Baigene S.L. (Alava Technology Park) under appropriate conditions (e.g., −80 °C) for up to 15 years, or indefinitely depending on the utility for research and while maintaining ethical approval and consent. Future use of these samples will require a new specific informed consent and the approval of the corresponding ethics committee for each project. All derived data will be handled in an anonymised or coded form.

### 7.4. Ancillary Care, Post-Trial and Compensation:

- Ancillary Care: Participants will continue with their usual medical care for any pre-existing or new conditions, independent of the trial, without participation in the study altering their access to health services.
- Post-Trial Care: After the end of participation (week 17), the IST intervention will not be continued as part of this protocol. General information on the importance of an active lifestyle and the benefits of strength training will be provided; continuation of exercise will be the responsibility of the individual or managed through health/community services.
- Compensation for Injury: If a participant suffers any injury as a direct result of their participation, they will receive appropriate medical treatment (see Section 2.4.3). No additional financial compensation beyond the coverage of the public health system or institutional insurance is foreseen. This will be reported in the informed consent.

## 8. Protocol Management and Dissemination

### 8.1. Protocol Modifications

Any substantial modifications to the protocol (e.g., changes in objectives, design, eligibility criteria, sample size, intervention, main outcomes or statistical analyses) will require a formal amendment. These will be proposed by the principal investigator, discussed with the research team and submitted for approval to the University of Deusto Research Ethics Committee prior to implementation. Major amendments will be communicated to relevant parties, including the investigator team, the clinical trials registry (updating information), trial participants (if their participation or consent is affected) and scientific journals at the time of publication (detailing deviations). A record of all protocol versions and amendments with their approval dates shall be maintained.

### 8.2. Access to Data

Access to the final, cleaned and anonymised trial dataset will be restricted to the principal investigator’s team, with the principal investigator (Iker López) being its custodian. Team members will have access to the data necessary for analyses and publications, according to their roles. There are no contractual agreements with the sponsor (University of Burgos) or collaborating organisations that limit the researchers’ access to the data or their right to publish them independently. Baigene S.L. will access the coded biological samples and raw genetic data for the agreed analyses; the principal investigator’s team will have full access to these processed data for integration, analysis and independent interpretation. Publications with genetic data will be co-authored with members of Baigene S.L. who have contributed intellectually, according to the authorship guidelines (see Section 8.3). Ownership of the raw data and main results will remain with the sponsoring institution and the principal investigator.

### 8.3. Data Dissemination, Authoring and Access Policy

- Communication of Results: The main results will be communicated to the scientific community through publication in peer-reviewed journals (seeking open access) and presentations at relevant conferences (geriatrics, sports medicine, exercise physiology, rehabilitation). Participants will be provided with an accessible summary of the main findings after publication. Avenues for communicating results to the general public and health professionals through institutional channels will be explored. There are no publication restrictions by the sponsor or collaborators.
- Authorship Guidelines: Authorship of publications will be determined according to the criteria of the International Committee of Medical Journal Editors (ICMJE). The use of professional editors is not foreseen.
- Access to the Protocol, Data and Statistical Code: This protocol will be made publicly available, ideally through publication. Following publication of the main results, sharing of the anonymised dataset and main statistical code will be considered, upon justified request and in accordance with institutional policies and data protection regulations, to promote transparency and reproducibility, while ensuring confidentiality.

## Figures and Tables

**Table 3:** Application Standards for IST Programme Exercises.

Exercise	Reference Figure	Main Muscle Group(s)	Positioning of the Participant	Key Joint Angle(s)	Instructions for Isometric Contraction	Key Monitoring Points/Common Pitfalls to Avoid
Peck Deck	a	Pectoralis major and anterior deltoid	Sitting on the CIEX machine with back against the backrest, 60° shoulder abduction, 60° horizontal shoulder adduction, 120° elbow flexion, forearms in neutral position and hands resting against the support.	*	Pushing the grips towards the centre of the body without movement, hold the contraction for the specified time in the three phases of progressive intensity while breathing continuously.	Avoid trunk offsets, do not perform the Valsalva manoeuvre, and ensure there is no visible joint movement.
Pull Over	b	Broad dorsal	Sitting on the CIEX machine with back against the backrest, shoulders bent at 90°, elbows resting on the supports and forearms in neutral position.	*	Push down on the supports without movement. Hold the contraction for the specified time in the three phases of progressive intensity while breathing continuously.	Avoid trunk flexion, prevent the back from moving away from the backrest, do not perform the Valsalva manoeuvre, and ensure that there is no visible joint movement.
Lateral Shoulder Elevation	c	Middle deltoid	Sitting on the CIEX machine with the back against the backrest and 90° abduction of the shoulders.	*	Push the supports up without movement, hold the contraction for the specified time in the three phases of progressive intensity while breathing continuously.	Avoid trunk compensations, avoid trapezius contractions, do not perform the Valsalva manoeuvre, and ensure that there is no visible joint movement.
Leg Extension	d	Quadriceps	Sitting on the CIEX machine with back against the backrest, hips flexed 90°, and knees flexed 90°.	*	Push the supports forward without movement, hold the contraction for the specified time in the three phases of progressive intensity while breathing continuously.	Avoid hip extension, avoid lumbar spine extension, do not perform the Valsalva manoeuvre, and ensure there is no visible joint movement.
Leg Curl	e	Hamstrings	Sitting on the CIEX machine with back against the backrest, hips flexed 90°, and knees flexed 90°.	*	Push the supports back without movement, hold the contraction for the specified time in the three phases of progressive intensity while breathing continuously.	Avoid hip flexion, avoid trunk flexion, do not perform the Valsalva manoeuvre, and ensure there is no visible joint movement.
Low Back	f	Square lumbar	Sitting on the CIEX machine with 60° hip flexion and shoulder blades resting on the support.	*	Push the support backwards, extending the lumbar spine without movement, hold the contraction for the specified time in the three phases of progressive intensity while breathing continuously.	Avoid trunk offsets, do not perform prolonged Valsalva manoeuvres, and ensure that there is no visible joint movement.
Hip Abduction	g	Gluteus maximus, gluteus medius and gluteus minimus.	Sitting on the CIEX machine with back against the backrest, with 45° hip extension, full knee extension and the inside of the feet resting on the supports.	*	Push the supports inwards without movement, hold the contraction for the specified time in the three phases of progressive intensity while breathing continuously.	Avoid hip and trunk extension, do not perform the Valsalva manoeuvre, and ensure there is no visible joint movement.
Hip Adduction	h	Adductor magnus, adductor magnus, adductor longus, pectineus and gracilis.	Sitting on the CIEX machine with back against the backrest, with 45° hip extension, 100° knee flexion and the outside of the knees resting on the supports.	*	Push the supports out without movement, hold the contraction for the specified time in the three phases of progressive intensity while breathing continuously.	Avoid hip and trunk flexion, do not perform prolonged Valsalva manoeuvre, ensure that there is no visible joint movement.

* Intermediate, determined individually according to the principles described in Section 2.1.2 (under ‘Determination of the specific angle’).

## Supplementary Materials

The following supporting information can be downloaded at: https://www.mdpi.com/article/10.3390/healthcare13131573/s1, Participant Information Sheet and Informed Consent Form Template; SPIRIT 2013 Checklist.
