# Supplementary material for: Protocol for a Trial to Assess the Efficacy and Applicability of Isometric Strength Training in Older Adults with Sarcopenia and Dynapenia
Source: Healthcare (Basel). 2025 Jul 1;13(13):1573. doi: 10.3390/healthcare13131573 (PMC12250102; doi:10.3390/healthcare13131573)
Supplement: Supplementary file 1 [file healthcare-13-01573-s001.zip › healthcare-3619820-supplementary.pdf]

12. Annexes

Annex A: Informed Consent Form Template

PARTICIPANT INFORMATION SHEET AND INFORMED CONSENT FORM

Study Title: Protocol for a trial to assess the efficacy and applicability of isometric strength training in older adults with sarcopenia and dynapenia.
Principal Investigator Iker López, *Licenciado en Ciencias de la Actividad Física y el Deporte* (BSc Physical Activity and Sports Science), Department of Health Sciences, Faculty of Health Sciences, University of Burgos, 09001 Burgos, Spain.
Sponsor Institution: University of Burgos
Collaborating Centres: University of Deusto, Kirolene, Baigene S.L., Vitoria-Gasteiz City Council (Judizmendi Civic Centre), Fundación Estadio Vital Fundazioa.

Dear participant,

You are invited to participate in a research study. Before deciding whether you wish to participate, it is important that you understand why the research is being conducted and what your participation will involve. Please take the time to read this information carefully and feel free to ask the research team any questions you may have.

1. What is the purpose of this study?

Ageing is often accompanied by a loss of muscle mass (sarcopenia) and a loss of strength (dynapenia), which can affect your ability to perform daily activities, increase the risk of falls and decrease your quality of life. Strength training is beneficial, but some traditional forms can be difficult or risky for older people. This study aims to assess whether a 16-week programme of Isometric Strength Training (IST) (a type of exercise where muscles are contracted without moving the joints) is effective in improving strength and muscle mass in adults over 70 years of age with sarcopenia and/or dynapenia. We also want to see if this type of training is safe, welcomed and easy for you to do, and how it influences your quality of life. In addition, we will explore whether certain genetic factors may influence how each person responds to training.

2. Why am I invited to participate?

You are being invited to participate because you are 70 years of age or older and, after an initial assessment, it has been determined that you meet the criteria for a diagnosis of sarcopenia (low muscle mass and/or low strength/physical performance), making you eligible for this study. Approximately 18 people like you will participate in this study.

3. If I decide to join the study, what will my participation involve?

If you decide to participate, your collaboration will last approximately 17-18 weeks and will involve the following:

- **Assessments (2 main sessions)**:
  - **Initial Assessment (Week 0, before training starts):** Will last approximately [Indicate estimated duration, e.g. 2-3 hours, could be split if too long]. It will include:
    - Questionnaires on your health, lifestyle, medication and quality of life (EuroQol EQ-5D-5L).
    - A brief geriatric assessment (functional, nutritional, cognitive and mood status).
    - Measurements of weight, height and Body Mass Index.
    - Analysis of your body composition (amount of muscle and fat) using bioimpedance (InBody 230), a non-invasive technique similar to stepping on a special scale.
    - Functional capacity tests: measurement of your walking speed, your hand grip strength, the maximum isometric strength of various muscle groups (legs, arms, trunk) on a specialised machine (CIEX SYSTEM) and an assessment of your posture using photographs.
    - Taking a saliva sample (by rubbing a swab on the inside of your cheek) for genetic analysis.
  - **Final Assessment (Week 17, one week after the end of the training):** Similar to the initial evaluation (except for the saliva sampling and genetic analysis, which is only done at the beginning), in order to see the changes. In addition, we will ask you a few questions about how confident and satisfied you feel, and how difficult you found the exercise programme.

- **Isometric Strength Training Programme**:
  - For **16 weeks**, you will attend **2 training sessions per week**, with at least 72 hours' rest in between.
  - Each session will last approximately **30 minutes**.
  - The sessions will be **individual and supervised** by a qualified personal trainer at the facilities of [Mention where they will be held, e.g. Kirolene / Judizmendi Civic Centre / Vital Stadium Foundation].
  - In each session, you will perform exercises for the main muscle groups on a specialised machine (CIEX SYSTEM) which will provide you with real-time feedback on the strength you are applying. The exercises consist of maintaining a muscle contraction without movement for a

given time, with an intensity that will increase progressively over the weeks.
    - You will be instructed on the correct technique, including proper breathing, for each exercise.

4. What are the possible risks or discomforts of participating?

Participation in this study involves minimal risk. IST is generally a safe form of exercise, especially for older adults, as it does not involve sudden joint movements.

- You may experience **temporary muscle fatigue or mild muscle soreness** after a few exercise sessions, similar to when you start a new physical activity. This discomfort is usually temporary.
- Assessment tests (such as strength or gait tests) are safe, but should be performed with caution to avoid discomfort. Saliva sampling is a simple and painless procedure. Bioimpedance testing is safe.
- During isometric strength exercises, your BP may increase momentarily. However, the protocol is designed with a gradual progression and you will be taught proper breathing to minimise this effect. In addition, people with uncontrolled hypertension will be excluded and their well-being will be monitored at each session. Any discomfort or adverse events you experience will be recorded and evaluated by the research team. If you suffer any harm as a direct result of your participation, you will receive appropriate medical treatment.

5. What are the potential benefits of participating?

- **Direct Benefits:** Although we cannot guarantee individual benefits, it is expected that participation in this IST programme may help you improve your muscle strength, and potentially your muscle mass and ability to perform daily activities. This could translate into a better quality of life. You will also receive a comprehensive assessment of your fitness level and body composition.

- **Indirect benefits:** Your participation will contribute to advancing researchers' understanding of how IST can help combat sarcopenia and dynapenia in older adults, and how genetic factors influence exercise response. This information could benefit other older people in the future and contribute to the development of more effective and personalised exercise programmes.

6. Are there alternatives to participation in this study?

Yes, your participation is completely voluntary. If you choose not to participate, or if you withdraw from the study at any time, you will continue to receive your usual medical attention and care without any harm to you.

7. Voluntary Participation and the Right to Withdraw:

Your participation in this study is **completely voluntary**. You may decide not to participate or to withdraw from the study at any time, without any explanation or penalty. This will not affect the medical care you receive or your relationship with the participating institutions in any way. If you withdraw, the data collected up to that point will be used, unless you indicate otherwise (provided that this is possible and the data have not already been anonymised for analysis).

8. Confidentiality and Protection of Personal Data:

All information collected about you during the survey will be treated as strictly confidential, in accordance with the General Data Protection Regulation (GDPR (EU) 2016/679) and applicable Spanish law. You will be assigned a numerical code to protect your identity. This code will be used in all study documents and databases. The list linking your name to the code will be stored securely and separately, with access restricted to the principal investigator team only. Saliva samples and genetic data will also be coded. The results of the study will be presented in an aggregated and anonymised form in scientific publications or at conferences, so that no individual participant can be identified.

9. Use of Biological Samples (Saliva) and Genetic Data:

Saliva samples will be used to extract their DNA and analyse certain genetic factors related to exercise response, sarcopenia and muscle ageing, in order to meet the objectives of this study.

- **[ ] I consent** to my biological samples (DNA) and derived genetic data being stored in encrypted form in the laboratory of Baigene S.L. (Álava Technology Park) for a period of up to 15 years (or indefinitely if it continues to be useful for ethically approved research) for possible use in **future research studies** related to sarcopenia, muscle ageing, response to exercise or other genetic analyses of scientific interest. I understand that this future use will require further approval by an ethics committee and that my data will continue to be treated confidentially.
- **[ ] I do not consent to** my biological (DNA) samples and derived genetic data being stored or used for future research studies beyond the scope of this study. I request that any leftover samples be destroyed upon completion of the analyses for this study.

Your decision about the future use of your samples will not affect your participation in the current study.

10. Compensation:

No financial compensation is offered for participation in this study. In the unlikely event that you suffer any harm directly related to your participation in the study, you will receive appropriate medical treatment. No other financial compensation for damages is foreseen.

11. Communication of Results:

If you wish, you can be informed of the overall results of the study once the study has been completed and the data have been analysed. You can also indicate whether you would like to know the results of your individual tests (e.g. body composition, strength), and they will be explained to you in an understandable way.

- [ ] I would like to know the results of my individual tests performed in the study.
- [ ] I do not wish to know the results of my individual tests performed in the study.
- [ ] I would like to receive a summary of the overall results of the study when they become available.

12. Additional Information and Contact:

If you have any questions about this study before, during or after your participation, or if you believe you have suffered any harm related to the study, you may contact the principal investigator:

- Iker López
- Department of Health Sciences, Faculty of Health Sciences, University of Burgos, 09001 Burgos, Spain
- ilt0002@alu.ubu.es

If you have questions about your rights as a participant in a research study, or if you wish to express any concerns or complaints about the study independently, you may contact the Research Ethics Committee of the University of Deusto:

- Arkaitz Castañeda-Babarro.
- Health, Physical Activity, and Sports Science Laboratory, Department of Physical Activity and Sports, Faculty of Education and Sport, University of Deusto, 48007 Bizkaia, Spain
- arkaitz.castaneda@deusto.es

PARTICIPANT'S DECLARATION OF CONSENT

I, Mr/Ms ...., with DNI/NIE ....,

- have read and understood the Participant Information Sheet (protocol version 2.0, dated 29 May 2025) for the study 'Protocol for a trial to evaluate the efficacy and applicability of isometric strength training in older adults with sarcopenia and dynapenia**'.**
- have had the opportunity to ask questions about the study and all my queries have been answered satisfactorily.
- understand that my participation is voluntary and that I may withdraw from the study at any time without explanation and without my medical care being affected in any way.
- voluntarily consent to participate in this study and to the use of my data (including genetic data) in an anonymised or coded form for the purposes of this research.
- (Concerning the future use of biological samples, I have marked my choice in paragraph 9).

- (Regarding being informed of my results, I have marked my choice in paragraph 11).

Signed in ...., on .... 20....

Participant's signature: Participant's name and surname:

Signature of Researcher (or person obtaining consent): Name and Surname:

(Two signed copies of this document will be provided, one for the participant and one for the researcher).

Annex B: SPIRIT 2013 Checklist - Locating Items in the Protocol

SPIRIT Item (No. and Short Description)	Location in article (Section)
**ADMINISTRATIVE INFORMATION**	
**1. Title:** Descriptive title identifying design, population, interventions and acronym (if applicable).	Main title of the document / Section 6.1.
**2a. Trial registration:** Trial identifier and registry name. If not yet registered, name of intended registry	Section 6.1 (Trial Registration).
**2b. Trial registration:** All items from the World Health Organization Trial Registration Data Set	Section 6.1 (Trial Registration).
**3. Protocol version:** Date and version identifier.	Section 6.1 (Protocol Version).
**4. Funding:** Sources and types of financial, material and other support.	Section 6.2 (Funding and Support).

**5a. Roles and responsibilities:** Names, affiliations and roles of contributors to the protocol.	Section 6.3 (Contributors to the Protocol) and title/authorship page.
**5b. Roles and responsibilities:** Name and contact information for the trial sponsor.	Section 6.3 (Trial Sponsor).
**5c. Roles and responsibilities:** Role of trial sponsor and funders in study design, collection, management, analysis, interpretation, drafting and decision to submit the report for publication.	Section 6.3 (Role of the Sponsor and Collaborating/Supporting Entities).
**5d. Roles and responsibilities:** Composition, roles and responsibilities of coordination/steering committees, etc. (if applicable).	Section 6.3 (Test Monitoring and Data Management).
**INTRODUCTION**	
**6a. Background and rationale:** Description of the research question, rationale for the trial, summary of relevant studies (benefits and harms of interventions).	Section 1 (INTRODUCTION).
**6b. Explanation for choice of comparators.**	Section 2.1 (Intervention Study Design).
**7. Objectives:** Specific objectives or hypotheses.	Section 1 (INTRODUCTION), at the end.
**8. Trial design:** Description of trial design, type of trial, allocation ratio, setting (superiority, exploratory, etc.).	Section 2.1 (Intervention Study Design).
**METHODS: Participants, interventions and outcomes**	
**9. Trial setting:** Description of settings and locations, list of countries. Reference to list of sites if extensive.	Section 2.1 (Intervention Study Design).

**10. Eligibility criteria:** Inclusion/exclusion criteria for participants. If applicable, for sites and individuals who will deliver the interventions.	Section 2.1.1 (Research Inclusion/Exclusion Criteria) and Section 2.1.3 (Research Staff Training).
**11a. Interventions:** Detailed description of interventions to enable replication (how and when).	Section 2.1.2 (IST Programme), Table 2 (IST Progression), (Figure 1 and Table 3 to be added).
**11b. Criteria for discontinuing/modifying interventions.**	Section 2.1.2 (Adherence to and Compliance with the Protocol -> Criteria for discontinuation or withdrawal of participants during the study).
**11c. Strategies to improve adherence and adherence monitoring.**	Section 2.1.2 (Adherence to and Protocol Compliance with the Protocol) and Section 2.1.3 (Monitoring Adherence, Compliance and Concomitant Variables).
**11d. Permitted/prohibited concomitant care and interventions.**	Section 2.1.2 (Adherence to and Compliance with the Protocol).
**12. Outcomes:** Primary, secondary, other; specific variable, analysis metrics, aggregation method, time point. Clinical relevance.	Section 2.2.1 (Study Outcome Variables).
**13. Participant timeline:** Timeline for recruitment, interventions, assessments, visits. Recommended timeline.	Section 2.1 (Intervention Study Design) - Table 1 (Study Participation Schedule).
**METHODS: Assignment of interventions (for controlled trials)**	
**16a. Sequence generation.**	Section 2.1.5 (Assignment of Intervention and Blinding -> Sequence Generation...).
**16b. Allocation concealment mechanism.**	Section 2.1.5 (Assignment of Intervention and Blinding -> Sequence Generation...).
**16c. Implementation of the assignment.**	Section 2.1.5 (Assignment of Intervention and Blinding -> Sequence Generation...).

**17a. Blinding (Masking):** Who will be blinded and how.	Section 2.1.5 (Blinding (Masking)).
**17b. Unblinding procedure (if blinding is present).**	Section 2.1.5 (Unblinding Procedure).
**METHODS: Data collection, management and analysis**	
**18a. Data collection methods:** Plans for assessment/data collection (outcome, baseline, other), quality processes, description of instruments (V&R if known), reference to CRF.	Sections 2.1.1, 2.2.2, 2.2.3. Section 2.1.3 (Control Mechanisms -> Training, Standardisation of Assessments and Minimisation of Biases; and Monitoring -> CRF sub-item). Section 2.2.2 ('Pre-intervention Assessment' for details on instruments).
**18b. Plans to promote retention and full follow-up, data to be collected if there is disruption/diversion.**	Section 2.1.3 (Strategies to Promote Participant Retention and Completeness of Follow-up). Section 2.1.2 (Discontinuation criteria -> final paragraph on data).
**19. Data management:** Plans for data entry, coding, security, storage; quality processes.	Section 2.1.4 (Data Management).
**20a. Statistical methods for primary and secondary outcomes.**	Section 2.3 (Statistical Analysis -> Analysis Strategy).
**20b. Methods for further analysis (subgroups, adjusted).**	Section 2.3 (Statistical Analysis -> Analysis Strategy).
**20c. Definition of analysis population (ITT, PP), handling of missing data.**	Section 2.3 (Statistical Analysis - beginning of section and paragraph on missing data).

**METHODS: Monitoring**	
**21a. Data Monitoring Committee (DMC):** Composition, role, independence. Alternatively, an explanation of why it is not needed.	Section 2.4.1 (Data Monitoring Committee (DMC)).
**21b. Interim analyses and trial stopping guidelines.**	Section 2.4.2 (Interim Analyses and Trial Stopping Guidelines).
**22. Harm (Adverse Events):** Plans for collecting, assessing, reporting and managing AEs.	Section 2.4.3 (Collection, Assessment, Reporting and Management of Adverse Events). Also Section 2.2.1 (Applicability of the IST protocol -> Safety).
**23. Audit:** Frequency and procedures for auditing the conduct of the trial, if any; independence.	Section 2.4.4 (Audit of Trial Conduct).
**ETHICS AND DISSEMINATION**	
**24. Research Ethics Approval:** Plans to seek REC approval.	Section 7.1 (Research Ethics Approval).
**25. Protocol amendments:** Plans for communicating major amendments to relevant parties.	Section 8.1 (Amendments to the Protocol).
**26a. Consent or assent:** Who will obtain consent and how.	Section 7.2 (Obtaining Primary Informed Consent), with reference to Section 2.1.1 (Stage One: Information and Consent).
**26b. Additional consent provisions for use of data/samples in ancillary studies.**	Section 7.2 (Consent for Ancillary Studies with Biological Samples), with reference to Section 2.1.1 (Consent for Ancillary Studies with Biological Samples).

**27. Confidentiality:** How personal information will be collected, shared and maintained to protect it.	Section 6.4 (Confidentiality and Data Protection).
**28. Declaration of interests:** Principal Investigators (PIs') financial and other competing interests.	Section 9 (DECLARATION OF CONFLICT OF INTEREST).
**29. Access to data:** Who will have access to the final dataset; contractual arrangements limiting access.	Section 8.2 (Data Access).
**30. Ancillary and post-trial care:** Provisions for care and compensation for damage.	Section 7.4 (Post-Trial Care and Compensation).
**31a. Dissemination policy:** Plans for communicating results to participants, professionals, public, etc.; publication restrictions.	Section 8.3 (Dissemination, Authoring and Data Access Policy -> Reporting of Results). Also Section 5 (CONCLUSION).
**31b. Dissemination policy:** Eligibility guidelines for authorship and use of professional editors.	Section 8.3 (Dissemination, Authoring and Data Access Policy -> Authoring Guidelines).
**31c. Dissemination policy:** Plans for public access to the protocol, participant-level data and statistical code.	Section 8.3 (Dissemination, Authoring and Data Access Policy -> Access to Protocol, Data and Statistical Code).
**ANNEXES**	
**32. Informed consent materials:** Sample consent form and other documentation.	Section 7.2 (Informed Consent Materials) and Annex A.
**33. Biological samples:** plans for collection, laboratory evaluation, storage and future use.	Section 7.3 (Biological Samples: Collection, Analysis, Storage and Future Use). Also Section 2.2.2 (Genetic Analysis).
